# Clinical factors predicting nemolizumab response in atopic dermatitis

**DOI:** 10.1016/j.jacig.2025.100457

**Published:** 2025-03-20

**Authors:** Yumi Masuo, Satoru Yonekura, Saeko Nakajima, Yu Sawada, Kenji Kabashima

**Affiliations:** a Department of Dermatology, Kyoto University Graduate School of Medicine, Kyoto, Japan; b Department of Drug Discovery for Inflammatory Skin Diseases, Kyoto University Graduate School of Medicine, Kyoto, Japan; c Department of Dermatology, Occupational and Environmental Medicine University Hospital, Fukuoka, Japan; d A∗STAR Skin Research Labs (A∗SRL), Agency for Science, Technology and Research (A∗STAR), Republic of Singapore; e Skin Research Institute of Singapore, Agency for Science, Technology and Research, Republic of Singapore

**Keywords:** Atopic dermatitis, nemolizumab, biomarker

## Abstract

Clinical predictors of response to nemolizumab, an anti-IL-31 receptor A antibody, were evaluated in patients with atopic dermatitis (AD). In a retrospective study of 14 Japanese patients, longer disease duration was significantly associated with poor response to treatment. While serum IgE levels and baseline Eczema Area Severity Index (EASI) scores alone were not predictive, their combination with disease duration improved discrimination between responders and nonresponders. Disease duration, serum IgE levels, and baseline EASI scores can be useful to predict nemolizumab efficacy in AD.

Nemolizumab is a humanized mAb targeting IL-31 receptor A, effectively reducing pruritus and decreased Eczema Area and Severity Index (EASI) scores by interrupting the itch-scratch cycles in atopic dermatitis (AD).^1^ However, real-world data reveal that not all patients with AD respond as well as expected, and new side effects have emerged since widespread use of nemolizumab in Japan began in 2022.^2^, ^3^, ^4^ Therefore, this study aimed to identify key indicators of nemolizumab efficacy by using clinical and laboratory data regarding patients with AD. The study retrospectively reviewed clinical records of patients with AD who were treated with nemolizumab between September 2022 and May 2024 at Kyoto University Hospital and Occupational and Environmental Medicine University Hospital. Those patients who achieved a reduction of 75% or more in their EASI score (EASI-75) and showed an improvement of 4 or more points on the Peak Pruritus Numeric Rating Scale (PP-NRS) after 3 cycles of nemolizumab were defined as responders,^5^ and those who did not meet these criteria were defined as nonresponders. Parameters of responders and nonresponders were compared by using the Mann-Whitney *U* test. Logistic regression analysis was performed using R software (version 4.2.2 [R Foundation for Statistical Computing, Vienna, Austria]) to discriminate between responders and nonresponders on the basis of parameter pairs. The decision boundary, representing the points at which the predicted probability was 0.5, was derived by setting the logit to zero in the logistic regression model. The resulting equation was transformed into a decision boundary formula that was visualized graphically to illustrate the separation between responders and nonresponders. All *P* values were 2 sided; *P* values less than .05 were considered statistically significant. This study followed the reporting guideline for case series.^6^

A total of 14 Japanese patients with AD were included in the analysis (Table I). Of the 14 case patients, 6 were classified as responders and 8 were classified as nonresponders. The nonresponders exhibited longer disease duration, higher serum IgE levels, and higher baseline EASI scores (Fig 1, *A-C*), with disease duration being the only parameter showing statistical significance between the groups. Other baseline parameters such as patient age, sex, systemic treatment before nemolizumab, baseline PP-NRS score, blood eosinophil percentage, serum thymus and activation-regulated chemokine (TARC) level, and serum lactate dehydrogenase (LDH) level showed no significant differences (Table I). After 3 treatment cycles, nonresponders trended toward increased serum LDH levels but not increased TARC or IgE levels (data not shown). Receiver operating characteristic (ROC) analysis was performed to assess each parameter's accuracy in distinguishing responders from nonresponders. Disease duration showed excellent predictive performance (area under the curve = 0.979 [Fig 1, *D*]), whereas serum IgE levels and baseline EASI scores demonstrated acceptable discriminative ability (area under the curve = 0.775 and 0.708, respectively [Fig 1, *E* and *F*]). The optimal cutoff values were as follows: disease duration, 16 years; serum IgE level, 240 IU/mL; and EASI score, 18.3. Given that single parameters other than disease duration showed limited discriminative ability, we performed logistic regression analyses to determine decision boundaries by using parameter combinations, aiming for more accurate discrimination between groups. The analyses revealed boundaries that effectively discriminated between responders and nonresponders in 3 parameter pair plots (Fig 1, *G-I* and see Table E1 in the Online Repository at www.jaci-global.org): EASI scores at baseline versus disease duration (correctly classifying 100% of responders and 100% of nonresponders [Fig 1, *G*]); disease duration versus IgE level (correctly classifying 100% of responders and 100% of nonresponders [Fig 1, *H*]); and EASI score at baseline versus IgE level (correctly classifying 60% of responders and 87.5% of nonresponders [Fig 1, *I*]). After nemolizumab failure, the treatment was switched to dupilumab. Symptom control was typically achieved within 2 to 3 months of switching therapy.

**Table I:** Baseline patient characteristics stratified by response to nemolizumab

Variables	Responder	Nonresponder	*P* value
No. of patients	6	8	
Age (y), median (range)	54.50 (16-76)	69.0 (37-85)	.547
Sex, no.			.209
Male	6	5	
Female	0	3	
Systemic treatment before nemolizumab			.194
None	2	4	
Cyclosporine	2	0	
Dupilumab	1	4	
Prednisolone	1	0	
PP-NRS score			
Blood eosinophils (%), median (range)	4.45 (1.1-10.6)	7.8 (2.1-10.4)	.414
LDH (IU/L), median (range)	246 (181-312)	208.5 (162-387)	.933
TARC (pg/mL), median (range)	1,831 (287.3-2,730)	971.7 (361-10,936)	.282

Patients who achieved a reduction of 75% or more in their EASI score and showed an improvement of 4 or more points in their PP-NRS score after 3 cycles of nemolizumab were defined as responders. Other baseline characteristics, including disease duration, EASI score, and PP-NRS score are shown in Fig 1, *A-C*. Continuous variables were compared by using the Mann-Whitney *U* test, and categoric variables were compared by using the using Fisher exact test.

**Fig 1:**
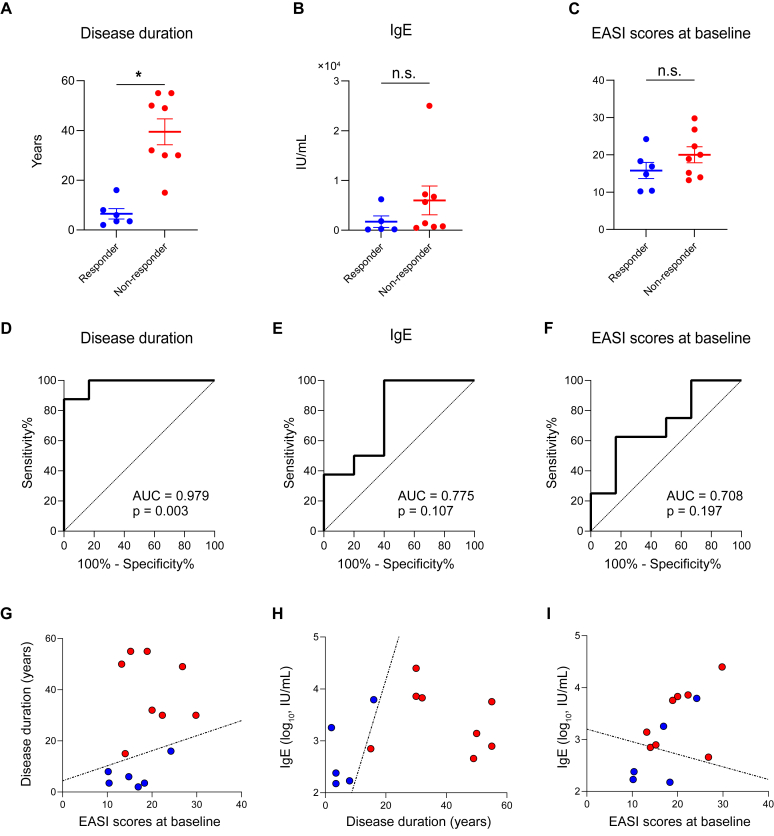
Clinical parameters between nemolizumab responders and nonresponders after 3 cycles. **A-C**, Baseline disease duration (**A**), IgE levels (**B**), and EASI scores (**C**). Horizontal bars represent mean values, with error bars indicating SEM. *P* values less than .05 are considered significant. **D-F**, Receiver operating characteristic analyses for optimal cutoff values. **G-I**, Scatter plots with responders (*blue*) and nonresponders (*red*) separated by decision boundaries. ∗*P* <.05. *AUC*, Area under curve.

Our study suggests that longer disease duration may predict a lack of response to nemolizumab, possibly because of exacerbated T_H_2 cell–mediated inflammation associated with IL-31 inhibition. In patients with AD, serum IgE levels correlate with T_H_2 cell–related inflammation and disease severity.^7^ In addition, chronic AD exhibits increased T_H_2 cell activity as well as other inflammation pathways.^8^ Interestingly, in murine models of allergic dermatitis, IL-31 deficiency was associated with increased IL-4–producing CD4^+^ T cells.^9^ Therefore, the observed unresponsiveness to nemolizumab in patients with AD may be attributed to a potential exacerbation of T_H_2 cell–mediated inflammation resulting from IL-31 inhibition. This inflammatory augmentation could potentially outweigh the beneficial effects on skin condition achieved through reduced itch-scratch cycles.

Although disease duration was the only parameter that showed statistically significant differences between responders and nonresponders, our logistic regression analysis demonstrated that EASI score and IgE levels could also contribute to discriminating between responders and nonresponders when analyzed in combination with the other parameters. These findings suggest that although individual parameters may not be sufficient predictors alone, their combined use could potentially enhance the ability to identify likely responders to nemolizumab. This suggests that even in clinical settings in which disease duration data may be unavailable or unreliable, the combination of available clinical parameters such as EASI index and IgE levels might still help to predict treatment response.

Several limitations of the study should be noted, including its retrospective design, small sample size, and lack of serum cytokine measurements, affecting the comparability and generalizability of our results. In particular, without IL-31 measurements, we could not directly validate the relationship between T_H_2 cell inflammation and treatment response. Future studies should include serum IL-31 levels to elucidate this potential mechanism. In addition, although logistic regression analysis revealed clear decision boundaries between responders and nonresponders, the regression coefficients did not reach statistical significance because of our small sample size. Therefore, these findings should be considered exploratory and warranting validation in larger cohorts.

In conclusion, disease duration, serum IgE levels, and baseline EASI scores can be useful to predict nemolizumab efficacy in patients with AD. Further studies are warranted to determine the most useful factors in the favorable response to nemolizumab among patients with AD.

## Disclosure statement

Disclosure of potential conflict of interest: S. Nakajima is a member of an industry-academia collaboration course with Maruho. K. Kabashima has received consulting fees from Maruho. Y. Sawada received the Takagi Award from Maruho Takagi Dermatology Foundation within the past 36 months. The rest of the authors declare that they have no relevant conflicts of interest.

## References

[1] Kabashima K., Matsumura T., Komazaki H., Kawashima M. (2020). Trial of nemolizumab and topical agents for atopic dermatitis with pruritus. N Engl J Med.

[2] Miyawaki K., Nakashima C., Otsuka A. (2023). Real-world effectiveness and safety of nemolizumab for the treatment of atopic dermatitis in Japanese patients: a single-centre retrospective study. Eur J Dermatol.

[3] Masuda T., Yonekura S., Kataoka K., Mizoguchi K., Hirata M., Fujimoto M. (2024). Psoriasis-like lesions in an atopic dermatitis patient possibly associated with nemolizumab treatment. J Dermatol.

[4] Masuyuki R., Sato E., Imafuku S. (2024). A case of bullous pemphigoid following administration of anti-IL-31 receptor A antibody. J Dermatol.

[5] Sidbury R., Alpizar S., Laquer V., Dhawan S., Abramovits W., Loprete L. (2022). Pharmacokinetics, safety, efficacy, and biomarker profiles during nemolizumab treatment of atopic dermatitis in adolescents. Dermatol Ther (Heidelb).

[6] Kempen J.H. (2011). Appropriate use and reporting of uncontrolled case series in the medical literature. Am J Ophthalmol.

[7] Uchida H., Kamata M., Nagata M., Fukaya S., Hayashi K., Fukuyasu A. (2020). Conjunctivitis in patients with atopic dermatitis treated with dupilumab is associated with higher baseline serum levels of immunoglobulin E and thymus and activation-regulated chemokine but not clinical severity in a real-world setting. J Am Acad Dermatol.

[8] Tsoi L.C., Rodriguez E., Stölzl D., Wehkamp U., Sun J., Gerdes S. (2020). Progression of acute-to-chronic atopic dermatitis is associated with quantitative rather than qualitative changes in cytokine responses. J Allergy Clin Immunol.

[9] Fassett M.S., Braz J.M., Castellanos C.A., Salvatierra J.J., Sadeghi M., Schroeder A.W. (2023). IL-31-dependent neurogenic inflammation restrains cutaneous type 2 immune response in allergic dermatitis. Sci Immunol.

