# Supplementary material for: Clinical factors predicting nemolizumab response in atopic dermatitis
Source: J Allergy Clin Immunol Glob. 2025 Mar 20;4(2):100457. doi: 10.1016/j.jacig.2025.100457 (PMC12002219; doi:10.1016/j.jacig.2025.100457)
Supplement: Supplementary Table 1 [file mmc1.docx]

**Supplementary Table 1. The decision boundary formulas**

| Parameter (x) | Parameter (y) | Formula |
| --- | --- | --- |
| EASI at baseline | Disease duration | y=0.588x + 4.39 |
| Disease duration | IgE (Log_10_) | y=0.1925x + 0.3247 |
| EASI at baseline | IgE (Log_10_) | y=-0.0242x + 3.2 |

**Supplementary Table 1 legend.** The decision boundary formula, representing the point where the predicted probability is 0.5, was derived by setting the logit to zero in the logistic regression model. The resulting equation was transformed into a decision boundary formula, which was visualized graphically to illustrate the separation between responders and non-responders. The regression coefficients did not reach statistical significance due to our small sample size. Therefore, these formulas should be considered exploratory.
